# Supplementary figures and images for: Comparative transcriptomics reveals new insights into melatonin-enhanced drought tolerance in naked oat seedlings
Source: PeerJ. 2022 Jun 28;10:e13669. doi: 10.7717/peerj.13669 (PMC9248784; doi:10.7717/peerj.13669)

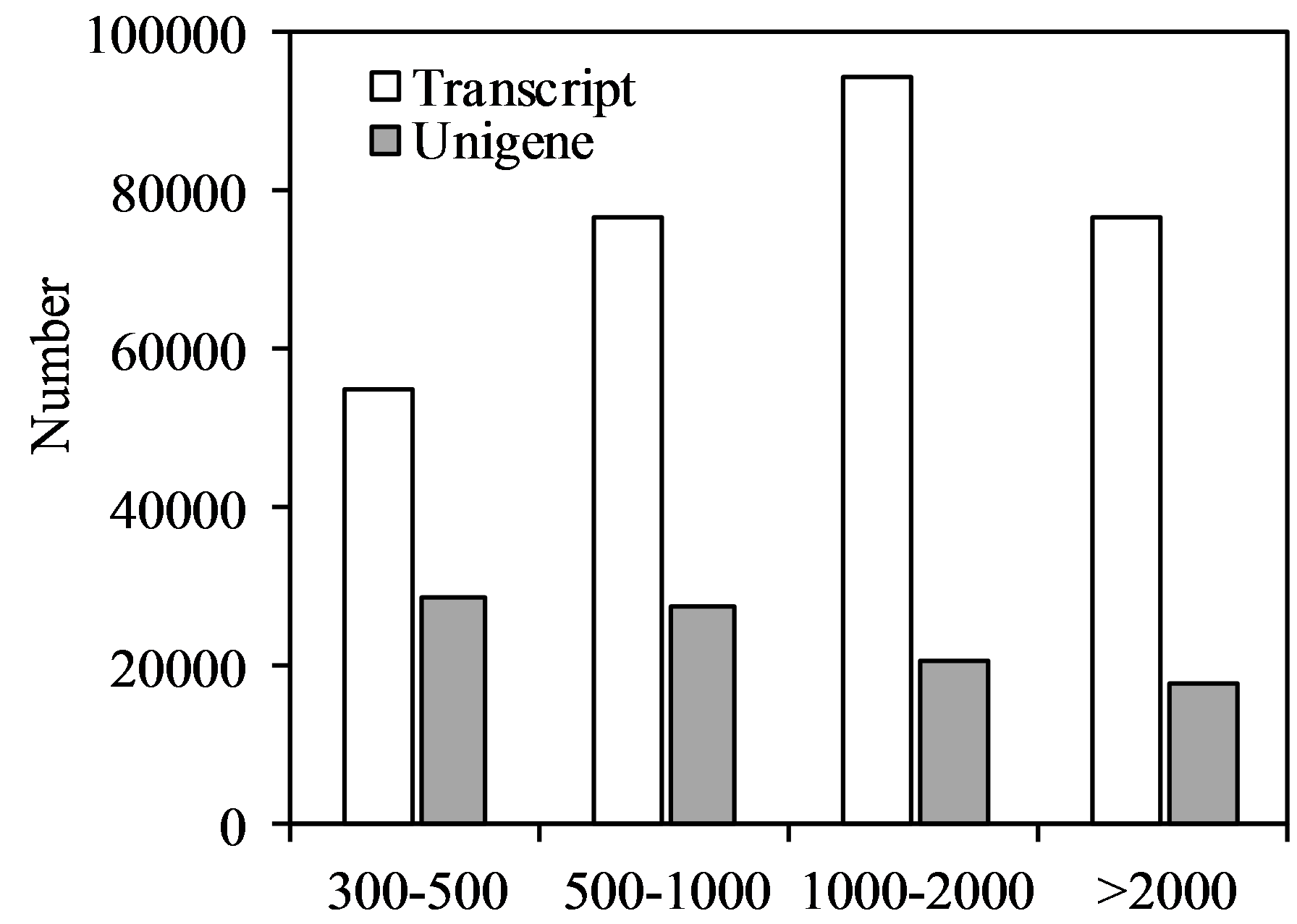

Supplement: Figure S1 [file peerj-10-13669-s001.png]

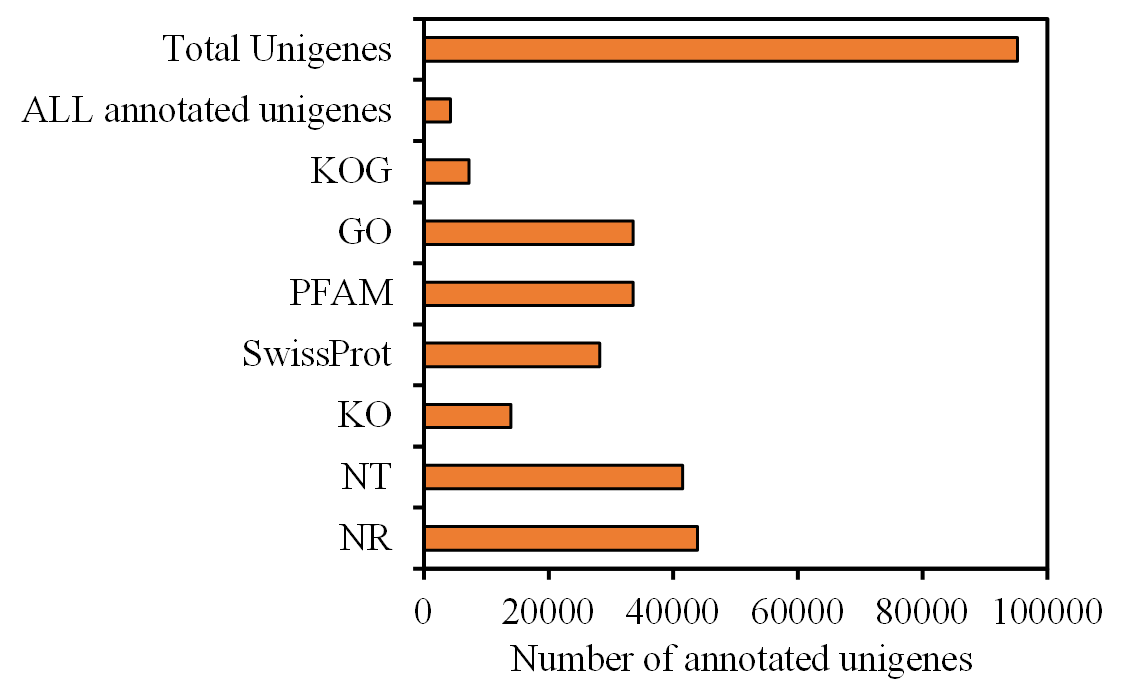

Supplement: Figure S2 [file peerj-10-13669-s002.png]

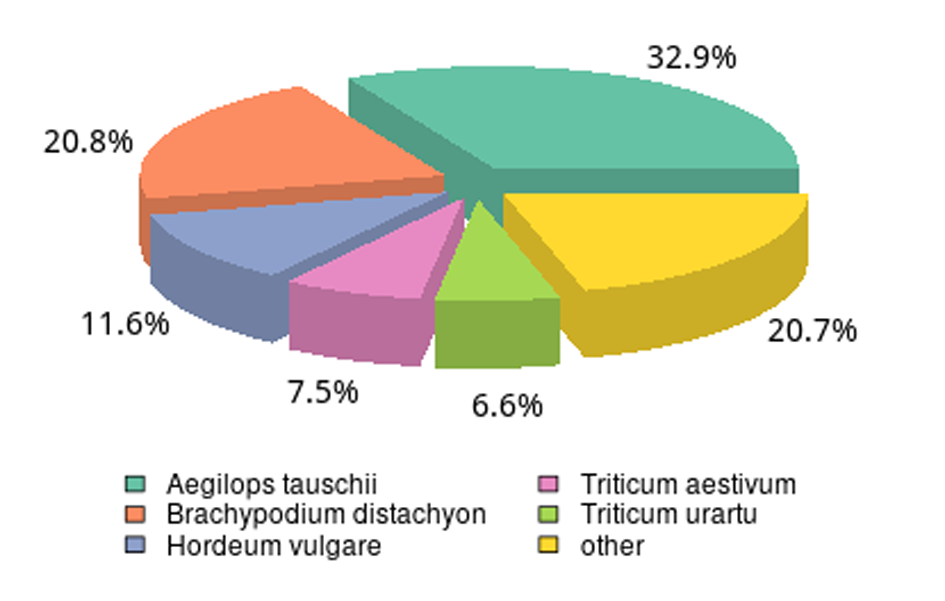

Supplement: Figure S3 [file peerj-10-13669-s003.png]

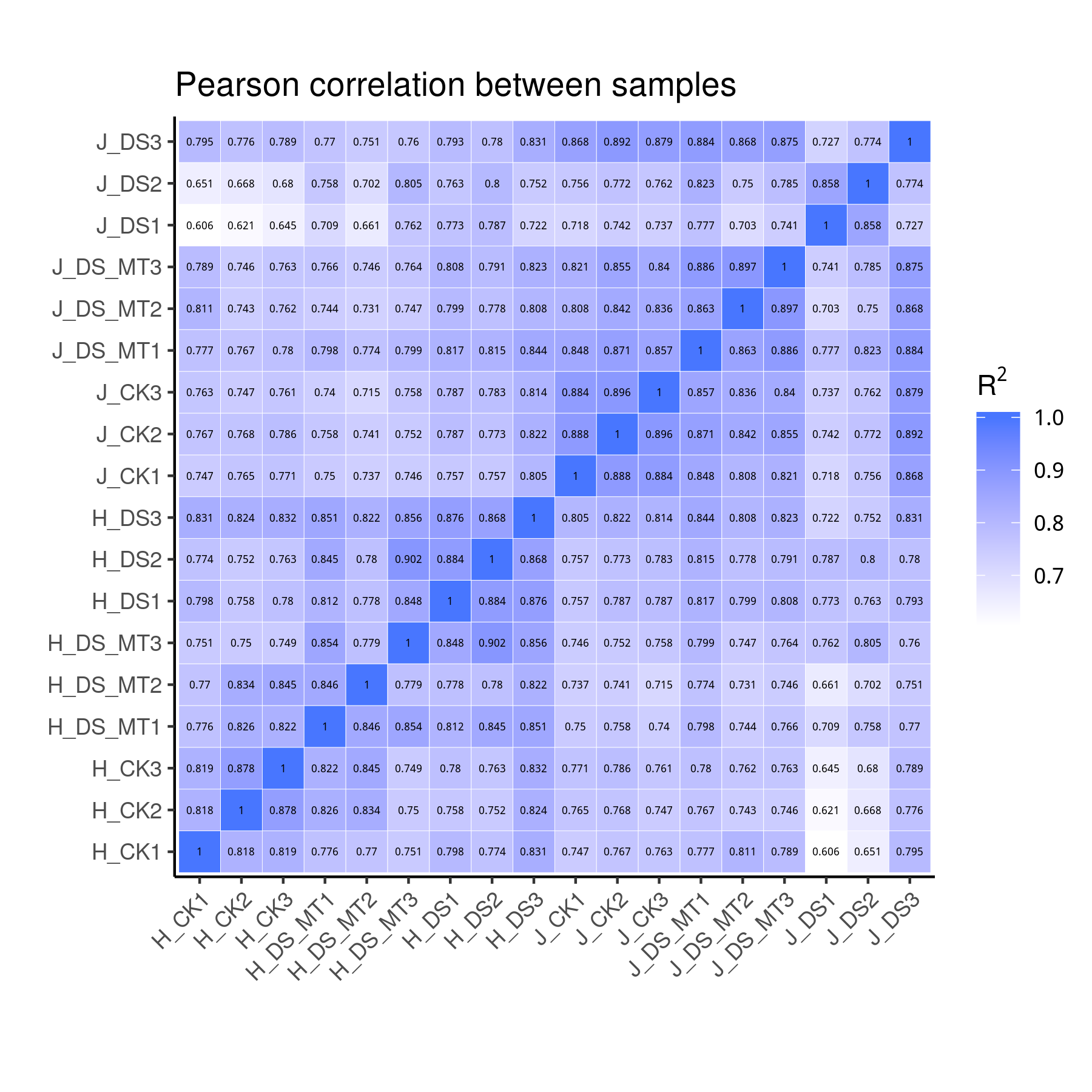

Supplement: Figure S4 — Each biological replicate of H2 is labeled as follows: CK were labeled H2_CK_1, H2_CK_2, H2_CK_3; DS were labeled H2_DS_1, H2_DS_2, H2_DS_3; DS+MT were labeled H2_DS+MT_1, H2_DS+MT_2, H2_DS+MT_3. Each biological replicate of J15 is labeled as follows: CK were labeled J15_CK_1, J15_CK_2, J15_CK_3; DS were labeled J15_DS_1, J15_DS_2, J15_DS_3; DS+MT were labeled J15_DS+MT_1, J15_DS+MT_2, J15_DS+MT_3. [file peerj-10-13669-s004.png]

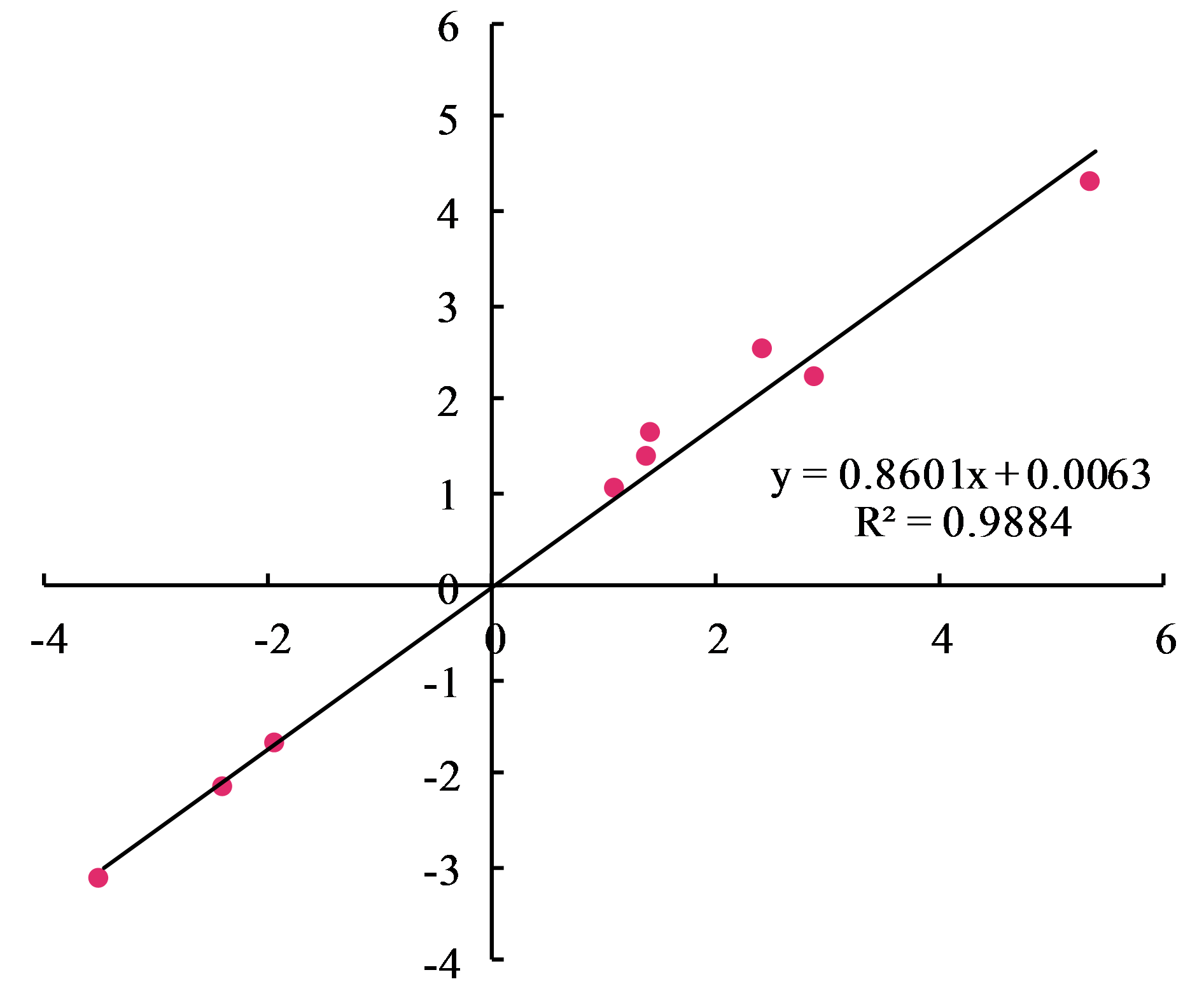

Supplement: Figure S5 [file peerj-10-13669-s005.png]
