# Supplementary material for: Comparative transcriptomics reveals new insights into melatonin-enhanced drought tolerance in naked oat seedlings
Source: PeerJ. 2022 Jun 28;10:e13669. doi: 10.7717/peerj.13669 (PMC9248784; doi:10.7717/peerj.13669)
Supplement: Table S1 [file peerj-10-13669-s006.docx]

## Table S2 Primers designed by qRT-PCR

| Gene name | Primer sequences (5′-3′) |
| --- | --- |
| Cluster-18670.50240 | F: GCCATTCGTGATACCCG  R: TCGTTCTGGGCTTCGTC |
| Cluster-18670.38545 | F: TCAGGCCGGGTTTCTT  R: CCATCACCTCCTTCCACA |
| Cluster-18670.30013 | F: CACTTAGCCCAATCCCTG  R: GCTTGTTCAATGGCATCA |
| Cluster-18670.32944 | F: GAATGAAGAGGTATGCGATGT  R: CAATAACCGTGATGTTGTCC |
| Cluster-18670.24664 | F: GTCATGTCCAACGGCTACG  R: TGCCACCTCAGTCTCCAAT |
| Cluster-18670.12661 | F: GTCGAGGAATAAACTCAGACG  R: TGGAACGCTTCAACAGGA |
| Cluster-18670.11860 | F: CTTTGAGATGAATGCGAGACCC  R: ACGCCCGTTTGCTGATCGATGTGCT |
| Cluster-18670.24627 | F: ACCTACGGCTACTGCTACG  R: AAACGCCTTCCAACCAC |
| Cluster-18670.41769 | F: GCAGAGCGCACCGCCAGCCATCCTG  R: CAGGATGGCTGGCGGTGCGCTCTGC |
