# Supplementary material for: Comparative transcriptomics reveals new insights into melatonin-enhanced drought tolerance in naked oat seedlings
Source: PeerJ. 2022 Jun 28;10:e13669. doi: 10.7717/peerj.13669 (PMC9248784; doi:10.7717/peerj.13669)
Supplement: Table S2 [file peerj-10-13669-s007.docx]

| Table S1 Overview of oats transcriptome sequencing data | | | | | |  |  |
| --- | --- | --- | --- | --- | --- | --- | --- |
| Sample | Raw reads | Clean reads | Clean bases | Error | Q20 (%) | Q30 (%) | GC (%) |
| H2_CK1 | 23455332 | 22546594 | 6.76G | 0.02 | 98.55 | 95.65 | 56.00 |
| H2_CK2 | 23181265 | 20779196 | 6.23G | 0.03 | 97.85 | 93.94 | 52.00 |
| H2_CK3 | 23137342 | 20946437 | 6.28G | 0.02 | 97.98 | 94.41 | 51.70 |
| H2_DS1 | 23384960 | 22738671 | 6.82G | 0.02 | 98.41 | 95.40 | 56.11 |
| H2_DS2 | 23587116 | 22853132 | 6.86G | 0.02 | 98.41 | 95.40 | 56.06 |
| H2_DS3 | 23381456 | 22833919 | 6.85G | 0.02 | 98.54 | 95.69 | 56.21 |
| H2_DS_MT1 | 26897614 | 26435734 | 7.93G | 0.03 | 97.80 | 93.66 | 53.79 |
| H2_DS_MT2 | 27205158 | 26786425 | 8.04G | 0.03 | 97.91 | 93.86 | 51.76 |
| H2_DS_MT3 | 25564215 | 24887073 | 7.47G | 0.02 | 98.46 | 95.50 | 56.17 |
| J15_CK1 | 22397306 | 21913550 | 6.57G | 0.02 | 98.53 | 95.61 | 55.30 |
| J15_CK2 | 23270973 | 22714579 | 6.81G | 0.02 | 98.50 | 95.55 | 55.38 |
| J15_CK3 | 22711258 | 22225906 | 6.67G | 0.02 | 98.29 | 95.09 | 56.83 |
| J15_DS1 | 22610962 | 21963509 | 6.59G | 0.02 | 98.60 | 95.73 | 53.63 |
| J15_DS2 | 23146871 | 22538398 | 6.76G | 0.02 | 98.55 | 95.67 | 54.33 |
| J15_DS3 | 22862392 | 22311437 | 6.69G | 0.02 | 98.50 | 95.56 | 56.13 |
| J15_DS_MT1 | 23479213 | 22782610 | 6.83G | 0.02 | 98.43 | 95.36 | 55.45 |
| J15_DS_MT2 | 22732857 | 22030550 | 6.61G | 0.03 | 97.56 | 93.38 | 56.20 |
| J15_DS_MT3 | 23945556 | 23173285 | 6.95G | 0.03 | 97.64 | 93.58 | 55.69 |
