# Supplementary material for: Comparative transcriptomics reveals new insights into melatonin-enhanced drought tolerance in naked oat seedlings
Source: PeerJ. 2022 Jun 28;10:e13669. doi: 10.7717/peerj.13669 (PMC9248784; doi:10.7717/peerj.13669)
Supplement: Table S3 [file peerj-10-13669-s008.docx]

## Table S3 Statistics for the oats transcriptome assembly

| Assembly | Total number | Total_nucleotides | Mean_length | N50 | N90 |
| --- | --- | --- | --- | --- | --- |
| Transcripts | 302,169 | 454,773,908 | 1,505 | 2,110 | 726 |
| Unigenes | 95,202 | 117,952,910 | 1,239 | 1,943 | 510 |
