# Supplementary material for: Comparative transcriptomics reveals new insights into melatonin-enhanced drought tolerance in naked oat seedlings
Source: PeerJ. 2022 Jun 28;10:e13669. doi: 10.7717/peerj.13669 (PMC9248784; doi:10.7717/peerj.13669)
Supplement: Table S6 [file peerj-10-13669-s011.docx]

## Table S6 Screening of 16 hormone-related genes involved in the response to melatonin supplementation under drought stress in naked oat seedlings from the DS+MT vs DS

| Gene ID | Gene name | log2FoldChange | Annotation |
| --- | --- | --- | --- |
| Cluster-18670.38166 | PYL | -3.01375 | Abscisic acid receptor PYL4 |
| Cluster-18670.50240 | PP2C | 4.92855 | Probable protein phosphatase 2C 30 |
| Cluster-18670.38545 | PP2C | 1.8416 | Probable protein phosphatase 2C 9 |
| Cluster-18670.32944 | PP2C | 2.02435 | Probable protein phosphatase 2C 6 |
| Cluster-18670.24664 | ABF | 2.6301 | bZIP transcription factor 23 |
| Cluster-18670.34242 | SNRK2 | 3.953 | Serine/threonine-protein kinase SAPK6 |
| Cluster-18670.10980 | IAA | 5.0596 | Auxin-responsive protein IAA9 |
| Cluster-18670.56105 | PP2C | 4.1206 | Probable protein phosphatase 2C 8 |
| Cluster-18670.40095 | SNRK2 | 3.15305 | Serine/threonine-protein kinase SAPK6 |
| Cluster-18670.57762 | PP2C | 9.9221 | Protein phosphatase 2C 51 |
| Cluster-18670.42950 | PP2C | 2.58325 | Protein phosphatase 2C 50 |
| Cluster-18670.43085 | PP2C | 2.48535 | Protein phosphatase 2C 50 |
| Cluster-18670.45168 | PP2C | 2.2713 | Protein phosphatase 2C 50 |
| Cluster-18670.45169 | PP2C | 2.91725 | Protein phosphatase 2C 50 |
| Cluster-18670.30764 | PP2C | 6.2353 | Probable protein phosphatase 2C 37 |
| Cluster-18670.13862 | PP2C | 5.01805 | Probable protein phosphatase 2C 37 |
| Cluster-18670.56630 | PP2C | 4.1694 | Probable protein phosphatase 2C 68 |
| Cluster-18670.35713 | PYL | -2.22065 | Abscisic acid receptor PYL4 |
