# Supplementary material for: Comparative transcriptomics reveals new insights into melatonin-enhanced drought tolerance in naked oat seedlings
Source: PeerJ. 2022 Jun 28;10:e13669. doi: 10.7717/peerj.13669 (PMC9248784; doi:10.7717/peerj.13669)
Supplement: Table S7 [file peerj-10-13669-s012.docx]

## Table S7 Screening of 4 nitrogen metabolism related genes involved in melatonin supplementation under drought stress in naked oats from the DS+MT vs DS

| Gene ID | Gene name | log2FoldChange | Annotation |
| --- | --- | --- | --- |
| Cluster-18670.11860 | cah | -2.7004 | Alpha carbonic anhydrase 7 |
| Cluster-18670.24627 | NRT, narK, nrtP, nasA | -1.8263 | Probable high-affinity nitrate transporter 2.4 |
| Cluster-18670.41769 | NR | -2.3393 | Nitrate reductase [NAD(P)H] |
| Cluster-18670.37627 | NR | -2.31565 | Nitrate reductase [NADH] |
